# Katalyst Pilot Study: Using Interactive Activities in Anatomy and Physiology to Teach Children the Scientific Foundation of Healthy Lifestyles

**DOI:** 10.3390/children5120162

**Published:** 2018-11-28

**Authors:** Rebecca L. Hagedorn, Kathryn Baker, Sara E. DeJarnett, Tyler Hendricks, Melissa McGowan, Lauren Joseph, Melissa D. Olfert

**Affiliations:** 1 Division of Animal and Nutritional Sciences, Davis College of Agriculture, Natural Resources & Design, West Virginia University, G025 Agricultural Science Building, Morgantown, WV 26506, USA; rlhagedorn@mix.wvu.edu (R.L.H.); sedejarnett@mix.wvu.edu (S.E.D.); 2 School of Medicine, West Virginia University, Morgantown, WV 26506, USA; kbaker17@mix.wvu.edu (K.B.); tah0017@mix.wvu.edu (T.H.); mpm0012@mix.wvu.edu (M.M.); lmj0003@mix.wvu.edu (L.J.)

**Keywords:** health education, lifestyles, experiential learning, students, elementary

## Abstract

This pilot study evaluated the impact of the Katalyst curriculum, a fifth-grade experiential learning program, on students’ knowledge of a healthy lifestyle’s impact on body functions. Katalyst’s interactive curriculum spans two days and includes four, 60-min stations on body systems: cardiovascular/endocrine, gastrointestinal, neurological, and respiratory/musculoskeletal. Three schools were recruited, and two schools completed the intervention sessions. Prior to beginning the stations, fifth-grade students completed a 37-item questionnaire to assess knowledge and perceptions. Students completed the same survey at the end of the Katalyst intervention. Teachers at the school also completed a survey post intervention to provide feedback on the program. Frequency and paired analyses were conducted on student responses and summative content analysis on teacher and volunteer feedback. The School 1 completer (*n* = 63) baseline mean knowledge score was 66.2%. The School 2 completer (*n* = 47) baseline mean knowledge score was 67.3%. Following the Katalyst intervention, both schools showed a statistically significant increase in the mean post score to 70.3% (*p* = 0.0017) and 78.4%(*p* < 0.0001) at School 1 (*n* = 63) and School 2 (*n* = 47), respectively. Teacher feedback (*n* = 7) revealed that Katalyst was effective in meeting state educational health standards and teachers perceived that the students benefitted from the program more than “reading about the body systems in a textbook or health magazine”. The Katalyst pilot study appeared to improve fifth-grade students’ knowledge of body systems and health. Katalyst aligned with state educational standards and is supported by teachers for an experiential learning opportunity. The Katalyst curriculum could be a potential avenue for health educators in Appalachia.

## 1. Introduction

Obesity has been a long-standing public health issue, with the national adult and youth obesity rates at 39.8% and 18.5%, respectively, and continuing to climb yearly [1,2,3]. The consequences of obesity on physical, mental, and emotional well-being have been well-studied and while obesity is detrimental at any age, obesity in youth is reported to cause long-term effects, including clinical issues such as hypertension, type 2 diabetes, inflammation, and sleep apnea, as well as self-esteem issues [4,5]. More specifically, studies have shown that children with obesity experience increased bullying among peers and have a higher risk of developing depression [6,7]. Obese youth are also more likely to become obese adults with higher morbidity and mortality rates [8]. Consequently, without intervention, weight gain and obesity will continue to cause long-term negative health effects. It is suggested that if the same lifestyle habits or environment as current times stay the same, national adult obesity and overweight prevalence will increase to 57.8% by 2030 [9]. This is of specific importance to regions of the United States that already tend to be more prone to health disparate issues, such as obesity. One region in particular, the Appalachian region, is home to some of the highest obesity rates, including West Virginia, which currently has the highest obesity rate at 38.1% [1].

To combat the childhood obesity epidemic, researchers have begun looking for public health approaches that target all children, instead of selective, risk factor approaches [10]. One potential pathway for broad childhood obesity prevention is intervention programs in schools [11,12,13,14]. Schools provide a means to target children from all socioeconomic, cultural, and racial backgrounds in an environment that is comfortable for children [11]. Additionally, schools are an integrable part of children’s lives, with children spending a significant portion of their childhood in a classroom, making it an ideal environment to target behavior [12]. Therefore, many studies have aimed to address healthy lifestyles and obesity prevention in the school setting, with varying success. Certain factors have been identified as essential for having the largest impact, including the curriculum components and teaching style. Specifically, experiential learning, or hands-on experience, is found to be one of the teaching strategies that shows the greatest effect on behavior change [15]. Additionally, curricula that are cross-curricular, including multiple topics into one lesson, are shown to be effective [15]. Therefore, it is essential when developing an intervention curriculum to include project-based and experiential learning that can include cross-curricular topics. Furthermore, incorporating curriculum topics that can coincide with state and federal curriculum standards can overcome barriers to school-based implementation and should be considered [16,17]. 

Within Appalachia, an aforementioned region of increased need that includes portions of 12 states and West Virginia in its entirety, childhood obesity prevention programs to promote healthy lifestyles are available, but limited, in school-based literature [18]. Those that are available fail to capitalize on the cross-curricular topics within education and mostly focus on nutrition education or physical activity. Specifically, incorporating science and health education together can capitalize on cross-curricular learning. This has been completed previously in “Health Is Life in Balance”, a hands-on diabetes curriculum that used an inquiry-based approach to not only educate students on how to live healthy lifestyles, but also to understand the sciences behind the decisions [19]. Following this example, programs developed for Appalachia should include education about multifaceted lifestyle behaviors to promote health (i.e., physical activity, nutrition, stress), but also inform on what healthy choices look like, why one should make them, and what happens to the body when healthy and unhealthy choices are made. To our knowledge, no curriculum in Appalachia incorporates interactive activities that involve anatomy and physiology to understand the scientific foundation of healthy lifestyles and educates on lifestyle behaviors to prevent chronic disease and obesity. Thus, to address this gap in available programming, the Katalyst curriculum was created and implemented. 

Katalyst is an experiential learning curriculum designed for fifth-grade students. The curriculum was specifically designed to address perceptions and behaviors that increase the risk of childhood obesity and educate on how to avoid these and apply healthy behaviors instead [20]. The curriculum utilizes Boud’s characteristics of experience-based learning [21] and allows students to participate in hands-on activities that educate them on a wide variety of health topics, including the cardiovascular/endocrine, gastrointestinal, neurological, and respiratory/musculoskeletal systems. Each system is taught to students in the classroom using an educational lesson about the organs that involves repetition of the content with facilitated discussions among students and leaders. Following the educational lesson, students engage in an experiential learning activity that demonstrates how the system works and what happens to these systems in a diseased state. This allows students to be hands-on and encompasses the fundamentals of experience-based learning through interactive games to engage the students’ senses and touch on topics relevant to the students’ lives. After the activities, students once again have a discussion with the station leader to incorporate reflective thinking and promote a deeper understanding of the topics. These different stations are designed to meet and implement the three objectives of the Katalyst study. The purpose of this project was to evaluate the effectiveness of the Katalyst school-based pilot intervention program in terms of increasing the knowledge of fifth-grade students through experiential learning and interactive lessons. Additionally, this manuscript evaluates fifth-grade teacher and Katalyst volunteers’ perceptions of the Katalyst program.

## 2. Materials and Methods

### 2.1. Design

This study was a pilot intervention of the Katalyst curriculum, an interactive fifth-grade curriculum to teach students the scientific foundation of healthy lifestyle behaviors in Appalachia. Katalyst sessions were led by trained student volunteers in the fifth-grade classrooms. A mixed-methods approach was implemented to gain a quantitative evaluation of fifth-grade student knowledge change and qualitative program feedback from teachers and college student volunteers. Data from fifth-grade students were collected at baseline before the Katalyst intervention and at the conclusion of the second day for post-test. Data from teachers and volunteers were collected at the conclusion of the Katalyst intervention sessions. The full Katalyst protocol and curriculum has been described previously [20]. This study was approved by West Virginia University’s Institutional Review Board #1703520227 to conduct an interventional program at elementary schools in Appalachia during the 2017-2018 academic year. An Application to Conduct Research was submitted and approved by the County Board of Education Committee to collaborate with local elementary schools.

### 2.2. Volunteer Recruitment and Participants

This study recruited graduate, professional, and undergraduate volunteers from the local university to lead the Katalyst intervention. The undergraduate students were designated as the primary teachers of each station’s material, with upper level graduate and professional students serving as mentors to the undergraduates in learning and teaching the material. Volunteers came from interdisciplinary programs, providing a broad base in knowledge in teaching the program to the fifth-grade students. 

Volunteers were recruited by emails sent through the health-related departments and through various student organizations. The advertisement included the aims of the program, details of the research project, volunteer requirements, and dates of the training meetings and school visits. A presentation was also given by Katalyst representatives at a premedical club meeting. 

Application forms were sent by email along with the advertisement. Students were required to be in good academic standing. Prior experience in a similar public outreach program was not required. The application form required educational information, employment details, references, four short answers, and a signed commitment to attend all of the training meetings and school visit dates if selected for the program. All students who applied by the deadline were invited for an in-person interview with a Katalyst representative. 

Twenty undergraduate students were selected to be volunteers, but eight students dropped out prior to the school visit, resulting in a final count of 12 undergraduate volunteers presenting at the pilot and intervention schools. Fourteen graduate and professional students participated in the program. The students represented a variety of majors, including Nutrition, Biology, Sports and Exercise Psychology, Biomedical Engineering, Psychology, and Exercise Physiology. 

### 2.3. Volunteer Training

Three 1.5-h mandatory training sessions were held for volunteers prior to running the Katalyst curriculum. The first training session began with an introduction to the program, including the goals and details of the program and schedule reminders. The volunteers were then split into four groups designated to each of the body systems. Graduate and professional students were permitted to choose their station, but the undergraduate students were randomly assigned to one of the four stations. Each station had approximately five undergraduate students, with three to four graduate and professional students. The protocol and the relevant PowerPoint Presentation were provided to each volunteer by email. The volunteers were assigned one or two concepts to teach at each station. The volunteers were taught to make each lesson interactive by asking questions and have the fifth-grade students repeat key concepts aloud. This was intended to not only keep the students engaged, but also to reinforce and ensure that the students were grasping the important information at each station. The graduate and professional students were called on to explain complex concepts related to the body systems and disease processes to the undergraduate students in order to enhance their ability to teach these concepts to the fifth-grade students. 

At the second training session, the volunteers rehearsed at their station and helped prepare teaching models and materials for the school visit. The third training session was dedicated to discussing issues faced during the Pilot Study and strategies to overcome these issues at the next school visit. 

### 2.4. School Recruitment and Participants

Elementary schools were recruited through invitation letters emailed to principals in local elementary schools. Schools that responded with interest were scheduled for in-person meetings between the authors and the principal of the school. During each meeting, the aims, methods, and procedures of this study were clarified, and dates were established for a visit based on schedule availability of the fifth-grade classes and the volunteers. Schools were offered a 50-dollar incentive for participating in the study. Of the eight local elementary schools contacted, three agreed to participate in the intervention. Unfortunately, one was scheduled for two days in March 2018 when an unforeseen state-wide teacher strike occurred, resulting in the cancellation of the school visit. The school had limited time available in the remaining academic year and was unable to reschedule the visit. Therefore, only two schools will be presented for completion of the intervention. 

All children in each participating school were invited to participate in the study. Parent consent forms were sent home with each fifth-grader two weeks prior to the school visit. The children were encouraged by their teachers to discuss the opportunity with their parents and return the signed forms if their parents were willing to let them participate. The consent form included the purpose of the study, a description of the procedures, risks and benefits, financial considerations, confidentiality, and reassurance of voluntary participation. Principals were encouraged to send a recorded call to the homes of children one week prior, encouraging parents to sign and return consent forms. Children who did not return the consent form, or those who had parents that declined consent, were still able to participate in the Katalyst lessons, but were excluded during data collection. Verbal assent was gained from children by researchers prior to the start of the study. In total, student consent and assent forms were gained from 74 and 49 students at School 1 (60% participation rate) and School 2 (53% participation rate), respectively.

### 2.5. Measures

Data collected in this study included knowledge questionnaires for the fifth-grade students and qualitative feedback from fifth-grade teachers. Data was collected electronically using Qualtrics^®^, an online survey platform. Fifth-grade students were provided with an identification (ID) number to use when filling out the survey to assess knowledge change from pre-post and used Chromebooks provided by their school to complete the survey. 

#### 2.5.1. Fifth-Grade Student Survey

Fifth-grade students completed a 37-item questionnaire at baseline and post intervention, using their assigned ID numbers. Pre-surveys were completed on the first day before starting instruction on any content and post-surveys were completed immediately following the second day of the curriculum. Content for the surveys was developed by the authors to coincide with the Katalyst curriculum. The multiple choice and true-false questions (*n* = 26) were specific to the information presented on the four body systems addressing knowledge of human physiology, the association between lifestyle behaviors and health, disease pathology, and practical knowledge in skills related to healthy lifestyle behaviors. Following the knowledge portion, students completed 12 questions using five-point Likert items (strongly disagree-strongly agree) questions assessing their perceptions of and attitudes towards healthy lifestyle behaviors, including the prevention of chronic diseases, their aptitude to adhere to healthy lifestyle behaviors, their interest in learning about the human body, and their interest in pursuing a career in a STEM (science, technology, engineering, mathematics) related field.

#### 2.5.2. Fifth-Grade Teacher’s Feedback Survey

Program feedback was grained from fifth-grade teachers through a seven-item open-answer questionnaire at the conclusion of each visit. Teachers were asked to evaluate program organization, volunteer preparation, station efficiency, lesson appropriateness, perception of benefits to fifth-grade students, and overall the delivered protocol for each station. Additionally, teachers were asked whether or not the Katalyst program met state educational standards or should be incorporated into the state’s standards. 

#### 2.5.3. Undergraduate and Medical Student Volunteers Survey

A 12-item questionnaire including Likert items (strongly disagree-strongly agree) and open-ended questions was provided to each volunteer at the conclusion of each school visit. The survey assessed the volunteers’ educational background, opinion on the importance of educating individuals on healthy lifestyle behaviors, the ability to prevent diseases through lifestyle behaviors, the efficacy of the Katalyst program, and their interest in continuing to participate in Katalyst. Additionally, it was assessed if their current collegiate program curriculum prepared them to discuss lifestyle behaviors with the public and if the Katalyst program benefitted their school curriculum. 

### 2.6. Statistical Analysis

Quantitative analyses were completed using JMP software (JMP^®^, Version Pro 12.2, SAS Institute Inc., Cary, NC, USA, 2015). Alpha was set at 0.05. Fifth-grade knowledge surveys were scored for correctness, resulting in a percent correct score for each student pre and post Katalyst. As there were only two classes, the usual options to accommodate this clustering were not appropriate and so analyses presented are stratified by school. McNemar’s test was used to compare each question for the knowledge survey baseline and post intervention. Signed Wilcoxon tests were used to assess the mean change in correct response from baseline to post intervention. Mann-Whitney U was used to analyze differences between School 1 and School 2 at baseline and post-test. Thematic analysis was conducted for qualitative data from teachers, with two researchers independently coding responses and both reviewers agreeing upon themes and sub-themes [16]. Both reviewers were trained in qualitative data analysis strategies. Frequency analysis was used to assess volunteers’ perceptions of the program.

## 3. Results

### 3.1. School Demographics

School 1 had a total enrollment of 846 students. The student population was 93% white and 22% of the student population received free and reduced lunch. School 2 had a total school enrollment of 665 students. The student population was 89% white and 41% of the student population received free and reduced lunch. These demographics do not specifically address the fifth-grade student population but provide a glance at the overall school environment. Student specific demographics were not collected.

### 3.2. Fifth Grade Student Knowledge

The School 1 baseline knowledge survey was completed by 74 students and School 2 completed by 49 students, with mean baseline scores of 66.2% and 67.0%, respectively. However, only students who completed baseline and post measures were considered completers and used for analysis. There was no significant difference between baseline completer scores at School 1 and School 2 (*p* = 0.3843). The School 1 completer (*n* = 63) baseline mean knowledge score was 66.2%. The School 2 completer (*n* = 47) baseline mean knowledge score was 67.3%. The change in correct response for each question on the knowledge test is available in Table 1. For School 1, the largest improvements in correct response were seen for “What does eating too much salt do?” (answer options: Prevents taking in oxygen in the air, Prevents taking in nutrients from our food, Lowers the amount of sugar in the blood, or Increases the pressure in blood vessels), with a 26% increase from baseline to post Katalyst intervention, and “How much exercise should 5th grade students get each day?” (answer options: 10 min each day, 20 min each day, 30 min each day, or 60 min each day), with a 20% increase from baseline to post Katalyst intervention. Questions at School 1 that saw a non-statistically significant decrease in correct response from baseline to post Katalyst intervention included “Which of the following cause obesity?” (answer options: High fat diets, High sugar diets, Not exercising, or All of the above), declining by 8%, and “What do the endocrine glands do?” (answer options: Send electrical signals to the heart, Release hormones, Attach to bones to allow movement, or Carry nutrients through the body), declining by 8%. School 2 saw the largest increase for “How much exercise should 5th grade students get each day?” (answer options: 10 min each day, 20 min each day, 30 min each day, or 60 min each day), with a 43% increase from baseline to post Katalyst intervention, and “Which option is healthier, whole-wheat bread or white rice”, with a 40% increase in correct response following the Katalyst intervention. Additionally, “Which nutrients are important for healthy bones” (answer options: Calcium, Vitamin D, Magnesium, or All of these are important for healthy bones) saw an increase of 31% in correct response from baseline to post intervention. A decrease in correct response for School 2, although non-statistically significant, was shown for the question “What disease involves clogged blood vessels? (answer options: Diabetes, Lung Disease, Drug Addiction, or Heart Disease), with a 3% decline, and “True or False: If parents are obese, then the child will become obese.”, with a 5% decline following the Katalyst intervention. Following the Katalyst intervention, both schools showed statistically significant increases in mean post score to 70.3% (*p* = 0.0017) and 78.4% (*p* < 0.0001) at School 1 (*n* = 63) and School 2 (*n* = 47), respectively. Compared to School 1, School 2 had significantly higher scores at post-test (*p* = 0.0001).

There were no significant improvements at either school regarding students’ perceptions. Changes from baseline to post intervention for School 1 and School 2 are shown in Table 2. Students at School 1 had a 5% and School 2 a 7% increase in agreeing or strongly agreeing that most diseases can be prevented through lifestyle habits. Additionally, students at School 1 had a 11% and School 2 had an 8% increase in stating they agree or strongly agree that they have the desire to eat less unhealthy foods to promote healthy habits. Students at both schools had high confidence in their abilities to choose a healthy meal, which improved by 2% and 6% from baseline to post intervention at School 1 and 2, respectively. Students found learning about the human body to be fun, with a 1% increase at School 1 and a 7% increase at School 2 from baseline to post intervention. Interest in STEM careers did not see a large increase at either school.

### 3.3. Teacher Feedback

The teacher survey was completed by eight teachers from School 1 (*n* = 5) and School 2 (*n* = 3). Two major themes were identified: benefit for students and room for improvement. Themes, supporting categories, and supporting quotes are shown in Table 3. All teachers stated they believed that Katalyst was a benefit to fifth-grade students’ education. When asked about the benefits for students, one teacher stated, “Seeing and interacting with materials is the best way for students to learn. This [Katalyst] is better than reading about the body systems in a textbook.” Another acknowledged that the fifth-grade students were able to interact with college students, stating, “Yes, this program [Katalyst] was a benefit. The [5th grade] students were given the opportunity to interact with college students for college readiness and continued the discussions on body systems even after the volunteers left the building.” Addressing lesson appropriateness, most teachers agreed that the topics were age appropriate and that the students could grasp the concepts. One teacher acknowledged that “Students were actively engaged in the lessons and lessons provided useful information in a way that 5th graders could understand as well as how that [health behaviors] can impact their bodies”. One teacher, however, identified that “some of the material may be a little too in depth for 5th graders”, specifically referencing the discussion on illegal drugs. When asked about Katalyst’s ability to meet state education standards, all teachers agreed that Katalyst covered topics that met many of the health standards for the fifth-grade curriculum. Teachers stated that Katalyst addressed many of the health standards that often get missed or fail to be covered due to the limited time to meet curriculum standards in a school year. Teachers were provided the opportunity to provide additional feedback on their perceptions of the program and anything that needed to be addressed in the curriculum. Again, teachers acknowledged that Katalyst students enjoyed being able to participate in Katalyst and “the students had continuing discussions even after the volunteers left the building”. Teachers had two recommendations to strengthen the Katalyst program focused on time and content. Teachers felt the curriculum could benefit from shortening the lessons from 2 hours to 1.5 hours to ensure that fifth-grade students can sustain attention and removing the discussion on illegal drugs.

### 3.4. Volunteer Perception

The volunteer survey was completed by 10 undergraduate students and seven graduate and professional students, who were predominately female (67%) and all, except one student, participated in both School 1 and School 2 visits. Graduate and professional (86%) and undergraduate (57%) students strongly agreed that healthy lifestyle habits can prevent most of the common diseases seen in patients and all volunteers (100%) strongly agreed that it is important to educate children on healthy lifestyle habits. When asked if they believed that Katalyst was successful in accomplishing its goals, 86% of graduate and professional students and 89% of undergraduate students strongly agreed or agreed that the program was successful. Both undergraduate and graduate/professional students expressed a desire to continue with Katalyst programming in the future. Assessment of Katalyst’s ability to benefit the volunteers’ current program curriculum showed that 71.4% of graduate/professional students and 78% of undergraduates believed that participating in Katalyst reinforced their current program curriculum and was a benefit to their learning as well. 

## 4. Discussion

This pilot study provides preliminary insight into the effectiveness and perceptions of the Katalyst program, a fifth-grade experiential learning health science curriculum. This pilot study provides an initial glimpse at Katalyst’s potential to improve fifth-grade students’ knowledge on the scientific foundation of a healthy lifestyle through hands-on anatomy and physiology activities. Two elementary schools participated in this pilot study, both of which saw a statistically significant increase in student knowledge scores from baseline to post-test. The students at these schools also displayed an increase in their interest to lead healthy lifestyles and the belief that chronic diseases can be prevented by their lifestyle choices, although these improvements were not statistically significant. Teachers and student volunteers believed that Katalyst was a successful program and teachers acknowledged Katalyst for its ability to benefit students beyond traditional education and meet state educational standards. 

As Appalachian populations tend to be more susceptible to poor lifestyle habits and chronic disease development, there is a need for evidence-based programming in this region [18,22]. There have been several successful programs throughout Appalachia that target different lifestyle behaviors, including sweetened-beverage consumption [23,24,25], physical activity [26,27,28], and dietary patterns [24,25,26,27,28,29,30]. The design of these programs varies in terms of the target age population and delivery method, but the majority tend to be in the third to fifth-grade age range and school-based. Similarly, Katalyst capitalized on a school-based approach with fifth-grade students. Specifically, within Appalachia, a majority of the population lives in remote, rural areas, making schools an important central hub for the delivery of health education programming that can reach all children in the area. 

The delivery of these school-based programs is majority teacher-driven. While teacher-driven programs can be successful, they also require time, interest, and additional training to implement a new curriculum that can create barriers to successful implementation [12,31]. An alternative model is through students as teachers. Within Appalachia, the use of high-school students to deliver lessons to younger students has been documented [29,30,32]. Using older students as mentors is noted as an innovative approach in promoting health behaviors of younger children and potential for health education in rural areas with limited funding for formal adult-mediated health-promotion programs [30]. In this way, Katalyst utilized partnership with local college student volunteers instead of relying on teacher-led education. This model was perceived as beneficial by the fifth-grade teachers, who mentioned that the fifth-grade students “were given an opportunity to interact with college students for college readiness” and it was also perceived as beneficial to the college student volunteers, who supported that Katalyst reinforced their college curriculum. This can provide a potential model for future programs to reduce teacher burden and capitalize on a cross-age teaching structure for benefits across multiple levels. 

While a few of these programs within Appalachia have assessed knowledge [24,27,30], this is predominantly limited to nutrition knowledge. To the best of our understanding, the investigation of health knowledge has only been conducted by one other program in the Appalachian region [24]. This intervention, also with fifth-grade students in the classroom setting, showed an increase in health knowledge after students participated in a four-session curriculum focused primarily on health factors for cardiovascular disease [24]. However, it is important to address more than cardiovascular disease as chronic diseases occur across multiple body systems. Therefore, Katalyst expands on previous health education programs by assessing student knowledge on not only the cardiovascular system, but the endocrine, gastrointestinal, neurological, respiratory, and musculoskeletal systems as well. 

The Katalyst study was strengthened by student volunteers that, except for one student, consistently attended Katalyst sessions to teach the curriculum. The use of the same volunteers may have contributed to the larger mean knowledge increase at School 2, as student volunteers learned and became comfortable presenting the curriculum. Although considered a success for showing the potential effectiveness of Katalyst for fifth-grade student science and health education, this pilot study is not without limitations. First, this study could have been strengthened by the addition of control schools. This was attempted, but following the teacher strike in West Virginia, no schools were willing to participate. However, the students in this study did not receive any significant health education at school between the pre and post assessments that could otherwise explain the improvement, suggesting that the changes were likely due to the Katalyst curriculum. Secondly, the school demographics are predominately White, limiting the generalizability of this program in more racially diverse elementary schools. Additionally, specific student demographics were not collected. This makes it unclear if demographic differences were present within or between schools. Additionally, this limits the understanding of any demographic differences between non-participating and participating students. Non-participating students were likely at random due to lost consent forms or busy parents, but some self-selection bias may have occurred, with more organized households and students returning consent forms to participate. Future studies should look to test the Katalyst curriculum in other areas in the Appalachian region, with the inclusion of specific student demographics. Next, the Katalyst surveys were not pulled from validated sources and should be tested for future use through test-retest methodology to ensure reliable results. The long-term outcome of student knowledge was also not assessed, which limits our knowledge of the impact of the Katalyst curriculum. Additionally, fifth-grade teachers acknowledged some limitations to the Katalyst curriculum. These limitations have been revised and an updated curriculum is ready for testing in other elementary classrooms. Lastly, this study provides initial results of the Katalyst study, but more long-term follow-up is needed to show if Katalyst can impact health behavior change and chronic disease prevention. 

## 5. Conclusions

Within Appalachia, and other health disparate regions, there is a need for programming that can promote healthy lifestyle choices to reduce chronic disease. This pilot study highlights the potential to improve fifth-grade student knowledge of the scientific foundation of healthy lifestyles through the Katalyst program, an experiential learning, anatomy- and physiology-based curriculum. The Katalyst curriculum has been revised based on participant feedback and is ready to be implemented in future school-based interventions in Appalachia. 

## Figures and Tables

**Table 1:** Knowledge survey correct response change from baseline to post intervention at school 1 and school 2.

Question	Correct Response	School 1			School 2		
		Baseline	Post	Change	Baseline	Post	Change
Which nutrients are important for healthy bones?	All of the above (Calcium, Vitamin D and Magnesium)	33%	46%	+13% *	39%	70%	+31% ***
Which meals allows us to build stronger muscles?	Foods high in protein	93%	98%	+5%	92%	94%	+2%
How much should 5th graders exercise each day?	60 min	72%	92%	+20% **	53%	96%	+43% ***
What does the brain do?	Sends nerve signals to control behavior, thoughts, emotion, and movement	94%	97%	+3%	92%	94%	+2%
What are nerves?	Connect the brain to the rest of the body	76%	84%	+8%	80%	85%	+5%
How do illegal drugs impact the body?	Prevent control over behavior	57%	64%	+7%	71%	87%	+16%
In what order does food move through the body?	Mouth, Esophagus, Stomach, Intestines	49%	65%	+16% *	71%	83%	+12%
What organ absorbs nutrients from food?	Intestines	60%	62%	+2%	67%	64%	−3%
Which of the following cause obesity?	All of the above (High-fat diets, High-sugar diets, Not exercising)	56%	48%	−8%	47%	70%	+23% *
What does the heart do?	Pumps blood throughout the body	93%	95%	+2%	92%	100%	+8%
What disease involved clogged blood vessels?	Heart Disease	57%	52%	−5%	63%	60%	−3%
Which of the following contributes to heart disease	All of the above (High fat and salt diets, Tobacco products, Not exercising)	44%	38%	−6%	31%	60%	+29% **
What does eating too much salt do?	Increases the pressure in the blood vessels	36%	62%	+26% **	46%	64%	+18% *
What do the lungs do?	Take in oxygen to breathe	97%	98%	+1%	88%	96%	+8%
What are the effects of tobacco products?	All of the above (Damage lungs, Damage blood vessels, Prevent the body from fighting illness)	22%	27%	+5%	29%	47%	+18% *
Which form of tobacco is safe to use?	No form of tobacco is safe	86%	92%	+6%	90%	96%	+6%
What do bones and muscles do?	Allow the body to move	79%	79%	-	84%	87%	+3%
True or False: If parents are obese, then the child will become obese.	False	78%	84%	+6%	90%	85%	−5%
Which would be the healthiest choice for breakfast?	Oatmeal with fruit	78%	79%	+1%	71%	77%	+6%
Which would be the healthiest choice for dinner?	Baked chicken, brown rice, steamed vegetables	54%	69%	+15% *	55%	68%	+13%
What would be a healthy choice for snack?	Fresh fruit	90%	91%	+1%	94%	92%	−2%
What do endocrine glands do?	Release hormones into the body	29%	21%	−8%	29%	30%	+1%
Which of the following involves too much sugar in the blood?	Diabetes	71%	84%	+13 *	76%	87%	+11%
What does sugar do?	Provides energy	75%	81%	+6%	80%	77%	−3%
Which food is a healthy source of sugar?	Fruit	72%	83%	+11%	69%	87%	+18 *
Which choice is healthier? Whole-wheat bread or white rice.	Whole-wheat bread	68%	86%	+18% **	45%	85%	+40% ***
Total Mean Knowledge Score		66.2%	70.3%	+4.1% **	67.3%	78.4%	+11.1% ***

* *p* < 0.05; ** *p* < 0.01; *** *p* < 0.001; McNemar’s test used to compare individual questions at baseline and post intervention. Signed Wilcoxon test used to compare mean knowledge score at baseline and post intervention.

**Table 2:** Student perception and attitudes change from baseline to post intervention at school 1 and school 2.

Statement	School 1			School 2		
	Baseline	Post	Change	Baseline	Post	Change
Most diseases can be prevented through healthy lifestyles	82%	87%	+5%	76%	83%	+7%
I am able to choose healthy meals	90%	92%	+2%	87%	93%	+6%
I want to eat less unhealthy foods for my health	79%	90%	+11%	76%	84%	+8%
Learning about the human body is fun	79%	80%	+1%	73%	80%	+7%
I am interested in careers that deal with STEM (science, technology, engineering, mathematics)	65%	65%	-	67%	68%	+1%

McNemar’s test used to compare statements from baseline and post intervention. Percentages represent students who strongly agree or agree with each statement.

**Table 3:** Thematic analysis results with associated quotes from fifth-grade school teachers.

Themes	Categories	Related Quotes
1 Benefit to students’	1.1 Experiential Learning 1.2 Fun, Age Appropriate Activities 1.3 Interaction with College Students 1.4 Covered Health Standards	Seeing and interacting with materials is the best way for students to learn. [Katalyst] is better than reading about the body systems in a textbook” “…lessons provided useful information in a way that 5th graders could understand as well as how that [health behaviors] can impact their bodies…” “The [5th grade] students were given the opportunity to interact with college students for college readiness…” “I loved the hands-on activities to model the body systems” The volunteers were prepared and interacted with the 5th grade students” “So great! [Katalyst] met many of the health standards we have not covered yet”
2 Room for Improvement	2.1 Length of Sessions 2.2 Depth of Content	“Students have a difficult time sustaining attention for two solid hours … recommend shortening the stations” “The stations were jam packed for two hours and the students wear out” “The content aligned with health standards but some material (referencing Hookahs, Meth, etc.) that was a little too in depth for 5th grade students”
